# Developing and validating a comprehensive measure of coordination in patient aligned care teams

**DOI:** 10.1186/s12913-022-08590-2

**Published:** 2022-10-10

**Authors:** Amber B. Amspoker, Houston F. Lester, Christiane Spitzmueller, Candice L. Thomas, Sylvia J. Hysong

**Affiliations:** 1 Center for Innovations in Quality, Effectiveness and Safety, Michael E. DeBakey VA Medical Center, 2002 Holcombe Blvd (152), 77030 Houston, TX USA; 2 Department of Medicine – Health Services Research Section, Baylor College of Medicine, Houston, TX USA; 3 Department of Management, University of Mississippi, Mississippi, USA; 4 Department of Psychology, University of Houston, Houston, TX USA; 5 Department of Psychology, St. Louis University, St. Louis, USA

**Keywords:** Coordination, Surveys and questionnaires, Primary health care

## Abstract

**Background/Objective:**

Despite numerous extant measures assessing context-specific elements of care coordination, we are unaware of any comprehensive, team-based instrument that measures the requisite mechanisms and conditions required to coordinate successfully. In this study we develop and validate the psychometric properties of the Coordination Practices Survey, a context-agnostic measure of coordination for primary care teams.

**Methods:**

Coordination items were developed based on a systematic literature review; items from previously developed scales were adapted and new items were created as needed; all items were refined after subject matter expert review and feedback. We collected data from Primary Care teams drawn from 1200 Veterans Health Administration (VHA) medical centers and outpatient clinics nationwide. 1645 primary care team members from 512 patient aligned care teams in the Veterans Health Administration completed the survey from 2015 to 2016. Psychometric properties were assessed after data collection using Cronbach’s alpha, intraclass correlations and multilevel confirmatory factor analysis to assess the factor structure.

**Results:**

Our findings confirmed the psychometric properties of two distinguishable subscales of coordination: (a) Accountability and (b) Common Understanding. The within- and between-team latent structure of each subscale exhibited adequate fit to the data, as well as appropriately high Cronbach’s alpha and intraclass correlations. There was insufficient variability in responses to the predictability subscale to properly assess its psychometric properties.

**Conclusion:**

With context-specific validation, our subscales of accountability and common understanding may be used to assess coordination processes in other contexts for both research and operational applications.

**Supplementary information:**

The online version contains supplementary material available at 10.1186/s12913-022-08590-2.

## Background

Coordination (the strategic sequencing of tasks amongst relevant parties to achieve a collective work) [1], whether amongst members of a healthcare team or between care delivery organizations, is a vital part of achieving desired healthcare outcomes. The National Academies of Science, Engineering and Medicine credit coordination with critical improvements in care quality, such as reducing fragmented care and duplicated effort, and avoiding diagnostic and treatment errors, thus allowing healthcare systems to function efficiently [2, 3]. Foundational to improving coordination, then, is our ability to measure it successfully.

Because coordinating healthcare (i.e., the purposeful organization of patient care activities to promote healthcare delivery) [4] involves multiple parties—such as providers and patients, different healthcare specialists, or members of interprofessional health care teams—numerous measures have been developed to assess specific elements of care coordination that vary depending upon context [5]. However, most attempts to measure and capture coordination in health care occur from the perspective of care coordination as an outcome, sometimes operationalized as specific healthcare-related tasks that were either completed or not completed [6]. Furthermore, despite existing frameworks explicitly highlighting the multiple parties involved in care coordination [7], it is often measured from a single perspective, for example, the referring provider, the patient, or the specialist [8]. Achieving the outcome of excellent care coordination, however, requires excelling at the process of coordinating–meaning all relevant parties involved in the coordinative act must be sampled [9]. Because existing measures are highly context- and role-specific, they often fall short of capturing the fundamental mechanisms and conditions required to successfully achieve a collective work [5, 9]. We are unaware of any comprehensive, team-based instrument that measures the requisite mechanisms and conditions required to coordinate successfully.

The lack of a comprehensive measure of team coordination is likely due to definitional ambiguity that manifests itself in two different ways: (1) differences amongst disciplines in the conceptualization of coordination, and (2) construct definitions that are too narrow, highly-context specific, and vary across studies even within a discipline. Thomas and colleagues demonstrated this problem in a literature review to determine availability of existing instruments of coordination (both inside and outside of healthcare), and called for an integrated measure that successfully assessed the fundamental mechanisms and conditions of coordination, divorced of clinical context [5].

### Study objective

In continuation of the work initiated by Thomas et al., the purpose of this manuscript is to develop a measure of coordination for teams and to assess its psychometric properties.

### Conceptual framework and instrument focus

To maintain consistency with Thomas et al.’s work, our proposed coordination instrument is based on Okhuysen and Bechky’s framework of coordination [1]. Okhuysen and Bechky’s framework synthesizes 30 years of coordination research from several fields, including models and frameworks known to the health care literature, such as Gittell’s relational coordination framework. Importantly, Okhuysen and Bechky’s coordination framework is process-centric and context agnostic – consequently it can be easily applied and (if needed) adapted to a wide variety of contexts, both in and outside of healthcare. It is also consistent with Weaver and colleagues’ Multiteam systems model of care coordination [10], which includes both Okhuysen and Bechky and the more well-known and healthcare-specific coordination framework from the Agency for Healthcare Research and Quality (AHRQ).

In their synthesis Okhuysen and Bechky identified five mechanisms (i.e., plans and rules, objects and representations, roles, routines, and proximity) that facilitate coordination and consist of many coordination processes (e.g., developing agreement and creating a common perspective). However, in attempting to create a general framework from these mechanisms, they found that many of the coordinating mechanisms can be substituted for one another and that coordinated behavior can be achieved as long as three integrating conditions are met: (1) *accountability* (knowing who is responsible for what), (2) *predictability* (knowing what tasks are involved and when they happen), and (3) *common understanding* (providing a shared perspective on the whole process and how individuals’ work fits within the whole). Consequently, in the interest of developing a comprehensive yet brief and usable coordination instrument that can be adopted in or adapted to multiple contexts, we focused on these integrative conditions that allow teams to coordinate.

## Methods

This paper is part of a larger study examining the impact of primary care team coordination practices on quality of care. A published protocol with detailed methods for the larger study is available elsewhere [9]. A brief overview of the coordination framework upon which the measure is based and the methods used to develop and validate the scale are presented here. The research reported here was reviewed and approved by the Baylor College of Medicine Institutional Review Board (protocol # H-30,952).

### Participants

2100 primary care Patient Aligned Care Teams (PACTs) from 152 U.S. Department of Veterans Affairs (VA) Medical Centers, Health Care Systems, 51 Primary Care Community-Based Outpatient Centers (CBOCs), and Multi-Specialty CBOCs, nationwide were randomly selected and invited to participate. All core members of each selected team (i.e., provider, nurse care manager, licensed vocational nurse, and scheduling clerk) were invited to participate. For purposes of construct validation, we opted to concentrate on the core PACT members as each team PACT is intended to have only one each of the aforementioned roles. Ancillary members (e.g., pharmacist, nutritionist, social worker), who are intended to service multiple teams and beyond by design, were excluded. Patients, who in many contexts are central elements of healthcare teams, were also excluded; as each PACT is responsible for the care of 1200 patients, including patients in the sample would have introduced dependencies in the data that would not have been feasible to parse in our analyses.

Invited PACTs were recruited via e-mail, which included a statement of informed consent and a link to the survey website. Those clicking on the link were directed to a page summarizing the aforementioned informed consent statement and indicating that by clicking “begin survey” they were providing their informed consent to participate.

### Measures: Survey development

The web-based Coordination Practices Survey was developed based on an extensive literature review of coordination within healthcare, psychology, and management; this review identified 279 survey items in 37 scientific articles (5 from within, 32 from outside healthcare) measuring various aspects of coordination [11]. Because of the central role that the integrating conditions play in coordination (see conceptual framework section, above), we focused on predictability, accountability, and common understanding. The literature review found no intact scales that measured the specific constructs in the Okhuysen and Bechky framework, finding instead scales that measured closely related constructs (e.g., team communication frequency, role clarity). Consequently, the team mapped individual items from these scales to the Okhuysen and Bechky constructs, and generated new items where needed (e.g., when there were not enough items to form a full scale) to construct a 15-item, context-free instrument of coordination practices that measure accountability, predictability, and common understanding [1]. Our team then adapted the items in this instrument to tailor them to the PACT context. For example, the following accountability item from the Thomas et al. instrument, “We have clearly established who in our team is responsible for particular aspects of a task“, was adapted for validation purposes as follows: “We have clearly established who in our PACT is responsible for particular aspects of a task.” Additional information regarding the items assessing coordination mechanisms and coordination processes is presented in Supplementary File 1. Once the items were adapted, an independent set of ten residents assigned to VA primary care clinics staff evaluated the survey for clarity, readability, and usability; item wording was then refined based on this evaluation. The process yielded five items each to measure predictability, accountability, and common understanding, respectively. Table 2 presents the 15 integrating condition items.

### Procedures: Survey deployment

To assess the psychometric properties of the Coordination Practices Survey, an online survey was distributed to members of clinical care teams within the VA Medical Centers. 2100 teams across all VA Medical Centers were randomly selected from approximately 5700 possible teams. We invited all core members of each selected team to complete an individual survey about their experiences with their team. If individuals were members of more than one team, they were asked to respond about the team that was randomly selected. Each respondent was asked to complete the survey only once. Reminder emails, messages, and phone calls were made to remind individuals to respond to the survey. To protect respondent confidentiality and to facilitate calculation of intraclass correlation coefficients, teams were only included when at least three individuals within the team completed the survey.

### Data analysis

Data were multilevel with employees nested within teams. We evaluated the applicability of Okhuysen & Bechky’s coordination model by first examining the variability and the intraclass correlation coefficient (ICC) for each item (i.e., the proportion of variance in each item that can be accounted for by variance between teams). As no widely-accepted standards are available for adequate within-team response rates for calculating ICCs, we drew from the work of Hirschfeld and colleagues [12] for guidance. Integrating conditions where many items had very low ICCs (i.e., < 0.05) or were skewed as indicated by a very small percentage (< 15%) of participants responding with “strongly disagree”, “disagree,” or “neither disagree nor agree” were evaluated to determine if items should be modified. We then conducted a multilevel confirmatory factor analysis (MCFA) for the integrating conditions using MPlus version 5.21.To assess goodness of fit of the model to the data, we examined the comparative fit index (CFI) and both the within- and between-team standardized root mean square residuals (SRMR). According to Kline [13], CFI values greater than 0.90 reflect good model fit. SRMR values below 0.05 indicate close fit, values around 0.08 indicate adequate fit, and values above 0.10 indicate poor fit [13, 14]. Chi-square (χ^2^)is also reported (with significant values indicating poor fit); it is considered a more useful means of comparing nested models than as an absolute indicator of model fit because, with large samples, it may be significant even when all other fit indices illustrate adequate fit [15].

Internal consistency reliability (Cronbach’s alpha) was then calculated for each integrating condition.

## Results

### Response rate and participant characteristics

**Table 1:** Final participant characteristics (n = 676, unless otherwise noted)

Characteristic	N (% of total)
Gender, N (%) (n = 605)	
Female	459 (75.87%)
Male	146 (24.13%)
PACT Role, N (%)	
Provider	159 (23.52%)
Care Manager	208 (30.77%)
Clinical Associate	168 (24.85%)
Clerical Associate	141 (20.86%)
Race/Ethnicity (n = 649)	
American Indian	6 (0.92%)
Asian	37 (5.70%)
Black	58 (8.94%)
Native Hawaiian	4 (0.62%)
Hispanic	66 (10.17%)
White	338 (52.08%)
Multiracial	14 (2.16%)
Other	7 (1.08%)
Prefer not to answer	119 (18.34%)
	**Mean (SD)**
Age (n = 595)	48.99 (10.45)
Number of years working for the VA (n = 653)	8.40 (7.91)
Number of years working with one’s current PACT (n = 653)	2.95 (2.34)

Note. Number of respondents differ from characteristic to characteristic because not everyone chose to answer all demographic questions. All available data are presented

300 primary care teams from our initial recruitment pool of 1200 had survey responses from 3 or more primary care personnel (for a total of 969 respondents), resulting in a 25% team-level response rate. Using more relaxed criteria, the team-level response rate for teams with responses from at least two members was 47%; for teams with responses from at least one member, the rate was 79%.

Due to low ICCs and negatively skewed responses, a subset of items were reworded (see item variability section, below) and the survey was redeployed to a new random sample of 900 teams. From the final sample of 900 primary care teams, 212 teams had survey responses from 3 or more primary care personnel (for a total of 676 respondents), resulting in a 23.56% team-level response rate. Of these 676 respondents, 159 (23.52%) were providers, 208 (30.76%) were registered nurse care managers, 168 (24.85%) were licensed practical nurses (clinical associates), and 141 (20.86%) were scheduling clerks. Average size of these teams was 3.25 members (SD = 0.49).

Table 1 summarizes participant characteristics of this final sample; Table 2 displays the items presented to said sample, which includes the final item set (see item variability section below for more details).

**Table 2:** Scale items and descriptive statistics for predictability, accountability, and common understanding

Item	n^†^	M	SD	ICC	% responded “strongly disagree,” “disagree,” or “neither”
Predictability*					
1. When a patient comes in for a visit, I have a good sense of all the tasks that should happen for the patient to receive well-coordinated care.	676	4.58	0.64	0.03	3.55%
2. For any given patient I can anticipate at what point in the sequence of the patient’s visit I am supposed to do my part.	671	4.62	0.64	0.04	3.58%
3. I always know the order in which my team members and I must do things to accomplish our goals.	673	4.45	0.78	0.06	8.32%
4. When working with the rest of my team, I am never uncertain of what our next steps are to move forward in our work.	672	4.17	1.01	0.07	16.96%
5. When caring for a patient, the set of tasks that need to be done to optimize patient care is clear to me.	673	4.45	0.79	0.05	7.58%
*Accountability*					
1. It is clear which team members in our PACT are responsible for completion of specific tasks.	674	4.27	0.91	0.13	12.17%
2. The division of responsibilities to complete a task is clear to all members of our PACT.	668	4.10	1.08	0.16	19.91%
3. Members of my PACT are able to hold each other accountable in making progress on joint tasks	674	4.06	1.08	0.19	21.36%
4. Specific responsibilities of each member of our team are transparent.	671	4.05	1.02	0.19	21.91%
5. We have clearly established who in our PACT is responsible for particular aspects of a task	674	4.13	0.99	0.19	17.80%
*Common Understanding*					
1. My PACT members and I are always on the same page about how our work fits with our organization	671	4.03	1.00	0.15	21.91%
2. Our team has a shared perspective of how each person’s work contributes to the overall goal of providing quality patient care.	675	4.19	0.94	0.17	15.85%
3. When it comes to providing patient care, my PACT members and I always share a common objective.	673	4.33	0.86	0.11	11.74%
4. My PACT members and I always share a common vision of how the work of taking care of a patient is supposed to unfold.	666	4.16	0.97	0.15	17.57%
5. When it comes to the care of the patient, everyone in my PACT is on the same page about “who on the team is supposed to do what when”.	670	4.07	1.03	0.20	22.24%

*Predictability items were not used in the MCFA due to inadequate ICCs and high negative skewness (see results section for more details). Reported statistics based on final sample of 212 teams consisting of 676 total participants. ^**†**^*n* reflects number of individual respondents. Items were rated on a 5-point Likert scale (1 = strongly disagree; 2 = disagree; 3 = neither agree nor disagree; 4 – agree; 5 = strongly agree)

### Item variability and ICCs

Among the initial sample, whereas most accountability items were normally distributed and exhibited ICCs of 0.05 or greater, 70% of the predictability and common understanding items were either negatively skewed (i.e., 85% or more of the responses were agree/strongly agree) or had a low percentage of between-team variance (i.e., ICCs < 0.05). Therefore, all 10 predictability and common understanding items were revisited and were reworded. We examined the distribution and ICCs for the new set of 15 integrating condition items in the final sample of 212 teams (consisting of 676 primary care personnel). Whereas 80% of the accountability and 80% of the new common understanding items demonstrated variability with ICCs greater than or equal to 0.05, several of the new predictability items still had ICCs < 0.05 and 80% had < 15% of participants who responded with “strongly disagree,” “disagree,” or “neither agree nor disagree.” Therefore, only the ten items used to measure accountability and common understanding were included in our subsequent MCFA, using data only from the second deployment of the survey.

### Assessment of latent scale structure

**Fig. 1:**
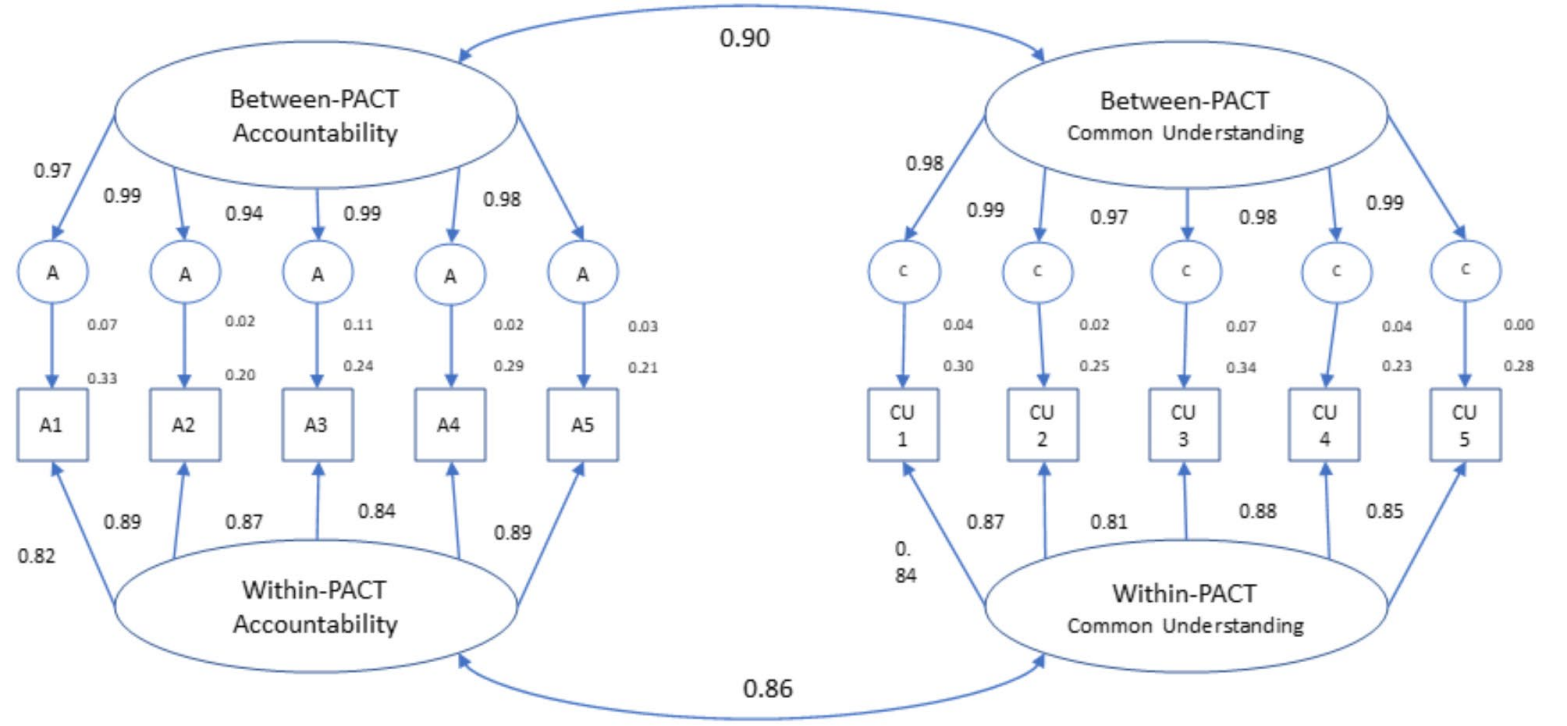
Results of Multilevel Confirmatory Factor Analysis for Accountability and Common Understanding Note. χ^2^ = 4335.45, p < 0.0001, CFI = 0.93, RMSEA = 0.08, SRMR within PACTs = 0.05, SRMR between PACTs = 0.06, Avg PACT ICC and range = 0.15 (0.10 to 0.20), indicating acceptable fit of the model.

The MCFA revealed that the two integrating conditions of common understanding and accountability were a good fit to the data, χ^2^ (df = 90, N = 676 respondents, N = 212 teams) = 4335.45, p < 0.0001, CFI = 0.93, SRMR within teams = 0.05, SRMR between teams = 0.06 (see Fig. 1). Factor loadings ranged between 0.94 and 0.99 for all items between-PACTs and between 0.81 and 0.89 for all items within-PACTs. Cronbach’s alpha was 0.93 for accountability and 0.94 for common understanding.

## Discussion

We developed and assessed the psychometric properties of a measure of team coordination agnostic to clinical condition, allowing it to be deployed in a wide variety of healthcare settings (e.g., primary care medical teams or any situation where team members are working together interdependently). The items in the instrument are sufficiently general that with minor modification of the referent in each item (e.g., from “our PACT” to “our team”), the items could be used in settings outside of healthcare, though the transportability of the scale’s psychometric properties would need to be confirmed.

We found support for two of the three integrating conditions. Specifically, the multilevel confirmatory factor analysis revealed that the model fit the data well for accountability and common understanding, and that all standardized factor loadings were larger than 0.80. As the predictability factor displayed very little between-team variability in our sample, we were unable to properly assess its fit. This lack of variability is likely due to the presence of centralized, national-level policies for delivering care to patients (e.g., test results must be released to patients within seven days; the ordering provider is responsible for following up with a patient with the results of the test), as well as the proceduralized nature of primary care work [16]. These two factors together create what is characterized as a strong situation [17]. Strong situations are construed similarly by participants (as evidenced by the lack of variability in our data), and induce uniform expectancies. These features of strong situations are highly consistent with the nature of primary care at the VA and what we observed in our data, and consistent with what has been found in other research [18]. In other samples where the situation is not as strong, sufficient variability in predictability responses may exist to allow for adequate assessment of the scale’s psychometrics.

### Limitations

As mentioned earlier, we were unable to properly assess the psychometric fitness of our predictability scale due to low variability in our sample. Although social desirability could account for such a response pattern, item wording for the other two scales was just as susceptible to social desirability bias, yet suffered no such problems of negative skewness or lack of variance. The nature of primary care work, as discussed earlier, is a more likely explanation. Another limitation is that many respondents work in more than one primary care team. Although they were instructed to respond from the perspective of a specific reference team, responses may not reflect their experience with the team where most of their time is spent, as the reference team was randomly selected by the researchers from the pool of teams to which a given respondent was assigned. This type of recall error would likely increase introduce unwanted error in item covariances and factor loadings.

Our instrument was validated on a national sample of primary care teams within the VA system, which is qualitatively different from fee-for-service primary care clinics, thus potentially limiting generalizability. However, significant variation exists in procedures and workflow from one VA medical center to the next [19]; thus, single-system bias could be mitigated by this variation. The instrument was also validated before the COVID-19 pandemic, which had considerable impact on workflows in primary care and related services. Nonetheless, the fundamental coordination constructs assessed by this instrument (Okhuysen and Bechky’s coordination processes and mechanisms), should not change because of the pandemic. Thus, although a team’s scores on a given scale could change, this would likely simply reflect a change in how the team decided to work together in their new reality, rather than any changes to the psychometric properties of the instrument.

Finally, team-level non-response statistics are not available, as we are not aware of any generally accepted standards for conducting team-level nonresponse. An individual-level non-response analyses was conducted as part of the larger study and reported elsewhere [20]. That analysis found statistically significant differences between survey respondents and non-respondents in age, FTE, and role (PCP vs. other). However, the magnitude of these differences was small (less than 5% points in any given characteristic), and unlikely to constitute a material source of bias in our analyses.

### Implications and future directions

Our instrument condenses a wide array of coordination constructs into two factors that can easily be administered for both research and operational needs. From a research perspective, team coordination is a process variable that can provide insight into why some teams may benefit from an intervention while others do not. Future research is needed to link our team coordination instrument to outcomes of interest. If this is found to be the case, an intervention designed to improve health outcomes (e.g., reduced duplication of effort) in team-based care settings may only attain those goals if the intervention first bolsters coordination before subsequently impacting the outcome of interest. Future research is also needed on the role of the patient in team coordination and the practicalities of its measurement.

From a practice perspective, our instrument can help decision-makers detect conditions that may explain why a quality improvement project did not have the desired effects on the outcome of interest, and also help identify and develop the conditions that foster effective coordination more broadly in their healthcare practices. Future improvement projects could attempt to strengthen the intervention-coordination relationship, which could yield subsequent outcome improvement.

## Conclusion

The number of different measurement approaches and collection of factors used to measure team coordination are evidence that it is perceived to be an important construct [5]. Okhuysen & Behcky [1] created a theoretical framework that encapsulates the many different implementations of coordination. We have developed a measure of team coordination that can be used for both research and operational purposes that can be used in a wide variety of contexts.

## Electronic supplementary material

Below is the link to the electronic supplementary material.

Supplementary Material 1

## Data Availability

The datasets generated during and/or analyzed during the current study are not publicly available as they contain information considered sensitive by the U.S. Department of Veterans Affairs (VA). Per VA policy, the data may be obtained via written request to the corresponding author, and released after approval from VA per current via regulations.
