# Supplementary material for: Developing and validating a comprehensive measure of coordination in patient aligned care teams
Source: BMC Health Serv Res. 2022 Oct 10;22:1243. doi: 10.1186/s12913-022-08590-2 (PMC9549451; doi:10.1186/s12913-022-08590-2)
Supplement: Supplementary file 1 — Supplementary Material 1 [file 12913_2022_8590_MOESM1_ESM.docx]

Appendix.  Multilevel Exploratory Factor Analysis Results for Coordinating Mechanism **Scales**

Okhusen and Bechky (2009) identified five mechanisms (e.g., plans and rules, objects and representations, roles, routines, and proximity) and three integrating conditions  (e.g., developing agreement and creating a common perspective) that facilitate coordinating behaviors. Although this work focuses on the 3 integrating conditions (i.e., *accountability,* *predictability, and common understanding)* that are more proximal to behavior, we also developed and evaluated items to measure each of the five coordinating mechanisms.  For reference, the items and results of multilevel exploratory factor analyses are included here.

***Table A1. Final Multilevel EFA for Plans and Rules***

|  | Within Teams | | Between Teams |
| --- | --- | --- | --- |
|  | Defining Responsibility for Tasks | Developing Agreement | Defining Responsibility for Tasks/Developing Agreement |
| There are concrete guidelines specifying the duties required of each  member of our PACT | **0.88** | -.09 | **0.99** |
| Our PACT has protocols for dividing responsibilities when there are gaps in staffing | **0.75** | 0.10 | **0.78** |
| Our PACT has specific guidelines that determine who is responsible for what aspects of patient care | **0.87** | 0.01 | **0.93** |
| In our PACT, we often review our approaches on addressing work related issues | 0.11 | **0.77** | **0.99** |
| Our team modifies our objectives in light of changing circumstances | 0.04 | **0.83** | **0.97** |
| Members of our PACT often discuss the methods used to get the job done | 0.00 | **0.90** | **0.96** |
| In our PACT, we talk to each other on  a regular basis to develop solutions to work problems | -0.17 | **0.98** | **1.00** |
| In our team meetings, we discuss and resolve necessary issues | 0.00 | **0.84** | **0.99** |
| Eigenvalues | 5.08 | 1.27 | 6.73 |

*Loadings that are bolded indicate simple structure (i.e., loadings of at least .60 on that are at least 2x as high on one factor than all other factors)

***Table A2. Final Multilevel EFA for Objects and Representations***

|  | Within PACTS | | | Between PACTS | | |
| --- | --- | --- | --- | --- | --- | --- |
|  | Direct Information Sharing | Scaffolding | Creating a Common Perspective | Scaffolding and Direct Information Sharing | Creating a Common Perspective | Scaffolding but under represented |
| PACT team members share necessary information to complete tasks | 0.16 | 0.57 | 0.04 | 0.37 | 0.58 | 0.13 |
| Our PACT has a common physical space where we can directly share crucial information to complete our work | **0.62** | 0.02 | 0.04 | **1.14** | 0.00 | -0.30 |
| PACT members can access the clinical information they need whenever they need it | **0.65** | 0.23 | -0.18 | **0.63** | -0.01 | 0.41 |
| Our PACT has shared tools to document important decisions | **0.74** | 0.09 | 0.01 | 0.24 | 0.47 | 0.41 |
| PACT members can easily share relevant information with other departments | **0.86** | -0.01 | -0.10 | 0.40 | -0.11 | 0.25 |
| Our PACT has a common virtual space where we can directly share crucial information to complete our work | **0.80** | -0.12 | 0.04 | **1.09** | 0.01 | -0.19 |
| Our PACT has visual aids that make our task progress visible to each other | 0.22 | 0.00 | **0.67** | 0.01 | 0.05 | **0.92** |
| We have systems and mechanisms (e.g., charts, white boards) that display team members’ progress on join tasks | 0.01 | -0.03 | **0.86** | 0.00 | 0.09 | **0.98** |
| Our PACT has tools to visualize what needs to get done on a given day | 0.02 | 0.08 | **0.83** | **0.72** | 0.33 | 0.05 |
| We have a concrete visual breakdown of who is responsible for what task | -0.01 | 0.15 | **0.72** | 0.53 | 0.62 | -0.02 |
| We have a shared understanding of how our PACT should operate on a daily basis | 0.01 | **0.78** | 0.07 | 0.16 | **0.83** | 0.13 |
| Everyone on the team has the same view of clinic workflows | 0.01 | **0.79** | 0.07 | -0.16 | **1.06** | -0.02 |
| Members of our PACT have a shared perspective on how to complete tasks related to patient care | -0.01 | **0.95** | -0.02 | -0.02 | **0.94** | 0.15 |
| Our PACT has a shared view of what needs to be done to complete our work | 0.00 | **0.95** | -0.02 | 0.28 | **0.83** | -0.01 |
| Eigenvalues | 7.21 | 1.84 | 1.09 | 9.12 | 1.75 | 1.15 |

*Loadings that are bolded indicate simple structure (i.e., loadings of at least .60 on that are at least 2x as high on one factor than all other factors)

***Table A3. Final Multilevel EFA for Routines***

|  | Within PACTs | | | Between PACTs | |
| --- | --- | --- | --- | --- | --- |
|  | Providing a template | Bringing Groups Together | Creating a Common Perspective | Providing a Template/CCP | Bringing groups together |
| There are established sequences of activities that help us track our progress when coordinating patient care | **0.69** | 0.17 | 0.01 | 0.33 | 0.73 |
| By following routines, we are able to know at any given point what steps are needed to complete a task | **0.76** | 0.04 | 0.08 | **0.96** | -0.07 |
| Those outside of our PACT can reasonably figure out where we are on a task because we follow a consistent routine | **0.91** | 0.22 | -0.05 | **0.92** | 0.04 |
| Our PACT has routine practices that allow us to make progress on a task even when circumstances change | **0.98** | -0.07 | -0.02 | **0.98** | 0.04 |
| We have routines that help us coordinate care without having to know every detail in advance | **0.87** | -0.06 | 0.05 | **0.97** | 0.04 |
| There are routine meetings within our PACT that enable us to work jointly on tasks | 0.22 | 0.29 | 0.35 | 0.26 | **0.86** |
| We meet regularly as a PACT to develop better ways to do our work | 0.16 | 0.33 | 0.36 | 0.24 | **0.87** |
| There are instances where our PACT meets with other groups outside of primary care to discuss better ways of doing our work | -0.01 | **0.91** | -0.01 | 0.00 | **0.99** |
| Our PACT meets routinely with other groups that rely on us, in order to jointly complete a task | 0.01 | **0.93** | 0.01 | -0.06 | **1.01** |
| Our facility provides regular opportunities (e.g., meetings) for our PACT to interact with other groups that rely on us | -0.01 | **0.85** | 0.00 | -0.12 | **0.88** |
| Our PACT members have a similar understanding of the treatment plans and goals for each of our patients | 0.14 | 0.00 | **0.71** | 0.63 | 0.54 |
| We have a shared understanding of how our PACT should operate on a daily basis | 0.09 | -0.02 | **0.78** | 0.76 | 0.41 |
| Everyone on the team has the same view of clinic workflows | 0.01 | 0.04 | **0.82** | **0.88** | -0.01 |
| Members of our PACT have a shared perspective on how to complete tasks related to patient care | -0.07 | 0.00 | **0.99** | **1.04** | -0.13 |
| Our PACT has a shared view of what needs to be done to complete our work | -0.03 | 0.00 | **0.96** | **1.00** | -0.02 |
| Eigenvalues | 8.73 | 1.72 | 1.12 | 11.55 | 1.74 |

*Loadings that are bolded indicate simple structure (i.e., loadings of at least .60 on that are at least 2x as high on one factor than all other factors)

***Table A4. Final Multilevel EFA for Roles***

|  | Within PACTs | | | Between PACTs | |
| --- | --- | --- | --- | --- | --- |
|  | Monitoring & Updating | Creating a Common Perspective | Role Substitution | Monitoring, CCP, and Role Sub | 4 items from Updating |
| In our team we carefully monitor each other’s work progress | **0.75** | 0.00 | 0.08 | **1.05** | -0.30 |
| The PACT leader closely monitors team members’ performance for errors | **0.87** | -0.01 | -0.07 | **0.98** | 0.01 |
| We have formal processes that allow us to observe each others’ work progress | **0.86** | 0.05 | -0.08 | **0.83** | 0.31 |
| In our team we check whether everyone is doing what is expected of them | **0.92** | -0.06 | -0.05 | **0.99** | 0.01 |
| The PACT leader tracks whether everyone completes their work | **0.93** | -0.08 | 0.00 | **0.88** | 0.22 |
| All team members report work-related progress to a supervisor | **0.75** | -0.02 | 0.06 | 0.07 | **0.97** |
| Our PACT provides work progress updates to other relevant parties | **0.68** | 0.08 | 0.08 | 0.00 | **1.00** |
| We provide informal progress updates to other PACTs or units we work with | **0.65** | 0.04 | 0.07 | 0.02 | **0.99** |
| We provide progress updates to our PACT leadership on a regular basis | **0.66** | 0.10 | 0.09 | -0.01 | **1.00** |
| Our PACT members have a similar understanding of the treatment plans and goals for each of our patients | 0.03 | **0.70** | 0.13 | 0.59 | 0.60 |
| We have a shared understanding of how our PACT should operate on a daily basis | 0.01 | **0.76** | 0.11 | **0.81** | 0.37 |
| Everyone on the team has the same view of clinic workflows | 0.09 | **0.83** | -0.05 | **0.89** | 0.03 |
| Members of our PACT have a shared perspective on how to complete tasks related to patient care | -0.09 | **1.00** | 0.00 | **0.97** | 0.06 |
| Our PACT has a shared view of what needs to be done to complete our work | -0.05 | **0.95** | 0.02 | **0.98** | 0.03 |
| Other members of my team are willing to help finish work that was assigned to me | -0.01 | 0.04 | **0.79** | **1.02** | -0.07 |
| In our PACT, we are willing to help other team members complete work even if it is not normally part of our job | -0.05 | 0.02 | **0.85** | **1.00** | -0.03 |
| In busy situations, members of our PACT have the necessary skills to help others on the team | 0.02 | -0.07 | **0.92** | 0.99 | 0.56 |
| My team members and I have the ability to help with each other’s work when necessary | 0.01 | 0.01 | **0.83** | 0.94 | 0.64 |
| Eigenvalues | 9.44 | 2.45 | 1.15 | 12.39 | 3.08 |

*Loadings that are bolded indicate simple structure (i.e., loadings of at least .60 on that are at least 2x as high on one factor than all other factors)

***Table A5. Final Multilevel EFA for Proximity***

|  | Within PACTs | | | Between PACTs | |
| --- | --- | --- | --- | --- | --- |
|  | Monitoring/Updating | Anticipating & Responding/Store of Knowledge | Developing Trust | Monitoring, updating, and SOK | A&R and DT |
| In our team we carefully monitor each other’s work progress | **0.75** | 0.12 | -0.01 | -0.45 | 0.96 |
| The PACT leader closely monitors team members’ performance for errors | **0.84** | 0.02 | -0.02 | **0.83** | 0.19 |
| We have formal processes that allow us to observe each others’ work progress | **0.85** | -0.01 | 0.05 | **0.73** | 0.28 |
| In our team we check whether everyone is doing what is expected of them | **0.91** | -0.04 | -0.02 | 0.54 | 0.55 |
| The PACT leader tracks whether everyone completes their work | **0.91** | -0.01 | -0.03 | **0.87** | 0.14 |
| Our PACT provides work progress updates to other relevant parties | **0.64** | 0.12 | 0.06 | **1.00** | -0.02 |
| We provide informal progress updates to other PACTs or units we work with | **0.63** | 0.13 | -0.01 | **1.00** | -0.10 |
| We provide progress updates to our PACT leadership on a regular basis | **0.63** | 0.19 | 0.03 | **1.02** | -0.07 |
| Members of our PACT effectively adapt their behavior to the actions of other members | 0.22 | 0.55 | 0.06 | 0.16 | **0.92** |
| Team members provide task-related information to each other without being asked | 0.18 | **0.60** | 0.13 | 0.06 | **0.97** |
| We anticipate each other’s actions and tailor what we do accordingly | 0.15 | **0.75** | 0.02 | -0.01 | **1.00** |
| Our PACT adjusts their behavior in anticipation of the actions of each other | 0.17 | **0.73** | -0.05 | 0.04 | **0.98** |
| Our team is aware of each other’s talents and skills | -0.10 | **0.99** | -0.07 | **0.86** | 0.21 |
| We learn about each other’s areas of expertise so we can rely on team members’ knowledge at the right time | -0.02 | **0.90** | 0.00 | **0.79** | 0.36 |
| In our PACT, we can easily determine who to approach to address a specific issue | 0.01 | **0.83** | 0.02 | 0.60 | 0.51 |
| We understand other team members’ expertise, and draw upon it when necessary | 0.00 | **0.85** | 0.02 | **0.79** | 0.36 |
| Members of our PACT approach their job with professionalism and dedication | 0.00 | 0.17 | **0.71** | 0.06 | **0.92** |
| Given my team’s track record, I see no reason to doubt the competence of my team members | 0.01 | 0.02 | **0.87** | 0.02 | **0.99** |
| When team members provide information, I do not feel the need to double check it | 0.01 | 0.01 | **0.79** | 0.03 | **0.98** |
| I have faith in my team members’ expertise | 0.00 | -0.01 | **0.97** | -0.05 | **1.02** |
| Our team members have the necessary knowledge and skill to perform well | 0.00 | 0.02 | **0.89** | -0.18 | **1.05** |
| I feel confident relying on the expertise that my team members bring to our PACT | -0.01 | -0.01 | **0.97** | -0.18 | **1.00** |
| Eigenvalues | 11.52 | 3.05 | 1.38 | 16.00 | 3.30 |

*Loadings that are bolded indicate simple structure (i.e., loadings of at least .60 on that are at least 2x as high on one factor than all other factors)
